# Burned-Out Testicular Tumor Presenting as a Retroperitoneal Mass: A Case Report

**DOI:** 10.7759/cureus.21603

**Published:** 2022-01-25

**Authors:** Soroush Shahrokh, Mohadese Shahin, Maryam Abolhasani, Amir Mohammad Arefpour

**Affiliations:** 1 Graduate Medical Education, HCA Houston Healthcare Kingwood/University of Houston School of Medicine, Kingwood, USA; 2 Department of Radiation Oncology, Faculty of Medicine, Iran University of Medical Sciences, Tehran, IRN; 3 Department of Pathology, Iran University of Medical Sciences, Tehran, IRN; 4 Department of Radiation Oncology, Iran University of Medical Sciences, Tehran, IRN

**Keywords:** retroperitoneal lymph node dissection, burned-out testicular tumor, burned-out tumor, germ cell tumor, testicular tumor, testicular cancer

## Abstract

Spontaneous primary tumor regression, or burned-out tumors, refers to the presence of a metastatic tumor with the histological regression of the primary lesion. The burned-out phenomenon has been reported in various malignancies, with testicular germ cell tumors (GCTs) accounting for a significant share of these cases. However, burned-out testicular tumors are a rare clinical phenomenon and are generally difficult to diagnose, as there is no evidence of primary testicular cancer. Here, we describe the case of a 42-year-old male who presented to our hospital complaining of right abdomen and groin pain for several months. On physical exam, the patient had normal genital and rectal exams. An abdominal-pelvic computed tomography (CT) scan of his abdomen and pelvis revealed a large retroperitoneal mass with radiographic characteristics of a sarcoma. Given his groin pain, the patient had a testicular ultrasound, which revealed scar tissue in the right testicle. His testicular tumor markers showed elevated β-human chorionic gonadotropin (β-hCG) and lactate dehydrogenase (LDH) but normal α-fetoprotein (AFP). He underwent right radical inguinal orchiectomy, with pathologic examination of the testicle revealing a burned-out testicular tumor. The patient was then treated with four cycles of bleomycin, etoposide, and cisplatin (BEP). His post-treatment tumor markers were normalized; however, his abdomen-pelvic CT scan showed a persistent mass. The patient underwent retroperitoneal lymph node dissection (RPLND) with the removal of 12 lymph nodes. However, pathologic evaluation of the lymph nodes revealed no evidence of neoplastic cells. The patient has remained disease-free after five years of follow-up.

This report highlights the potential of burned-out testicular tumors in young and middle-aged men presenting with a retroperitoneal mass. Furthermore, it underscores the importance of obtaining testicular ultrasound in these patients to rule out regressed testicular tumors.

## Introduction

Testicular cancer is a rare malignancy, accounting for only 1% of all malignant tumors in men [1]. However, it is the most common cancer in men aged 15-40 years old, with the global incidence steadily rising [2]. About 95% of testicular cancers are germ cell tumors (GCTs), divided into seminoma and non-seminoma GCTs (NSGCTs) [3]. NSGCTs include teratomas, yolk sac tumors, embryonal carcinomas, choriocarcinomas, and mixed GCTs [3]. While generally aggressive, testicular GCTs have an excellent prognosis with an approximately 90% five-year survival rate [4]. Treatment involves radical orchiectomy and a combination of chemotherapy, radiotherapy, and retroperitoneal lymph node dissection (RPLND), depending on the tumor stage and histology [4]. Spontaneous primary tumor regression, or burned-out tumors, has been reported in various malignancies, with testicular GCTs accounting for a significant share of these cases [5-9].

Here, we present the case of a 42-year-old male with a large retroperitoneal mass that was initially suspected to be a primary retroperitoneal sarcoma but later revealed to be metastasis from a burned-out testicular tumor.

## Case presentation

A 42-year-old male with no significant medical history presented to our hospital complaining of progressively worsening dull pain in his right lower abdomen, groin, and thigh for the past six months. His physical exam revealed diffuse lower abdominal and right groin tenderness, with mild right groin and thigh swelling. His rectal and genital exams were normal. Color doppler ultrasound of his right lower extremity showed normal blood flow with no evidence of thrombosis. 

**Figure 1:**
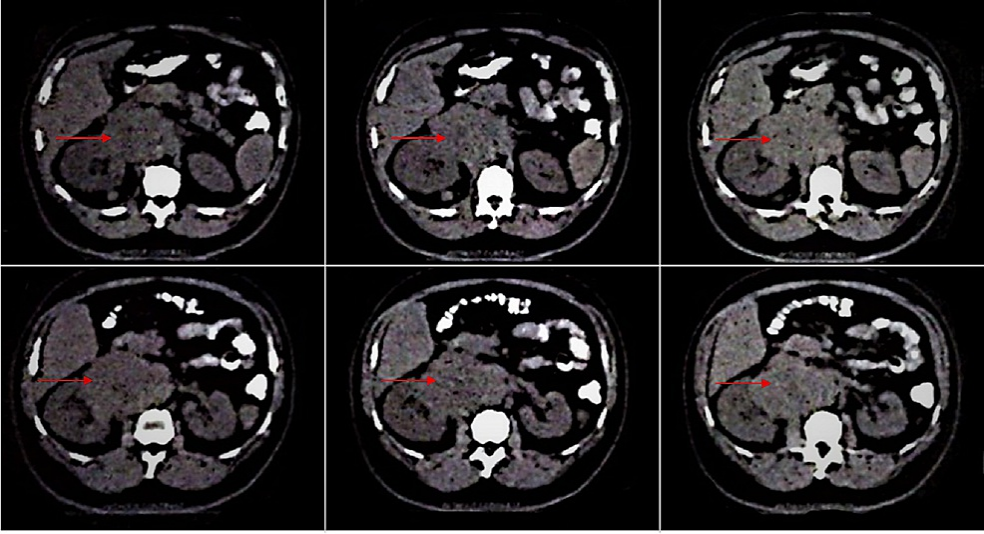
Initial CT-scan of the abdomen and pelvis prior to starting chemotherapy showed a large right retroperitoneal mass causing lateral displacement of the kidney. CT: computed tomography. Red arrows point to the retroperitoneal tumor.

**Figure 2:**
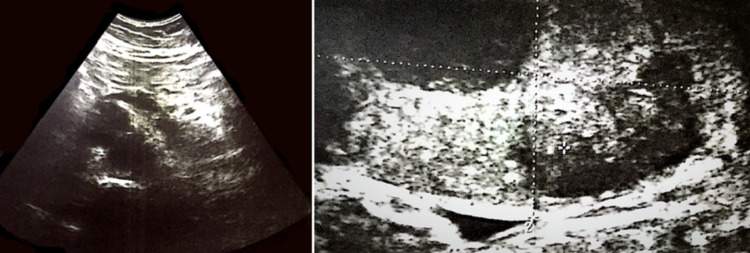
Testicular ultrasound showed two hypoechoic nodules in the right testis.

**Figure 3:**
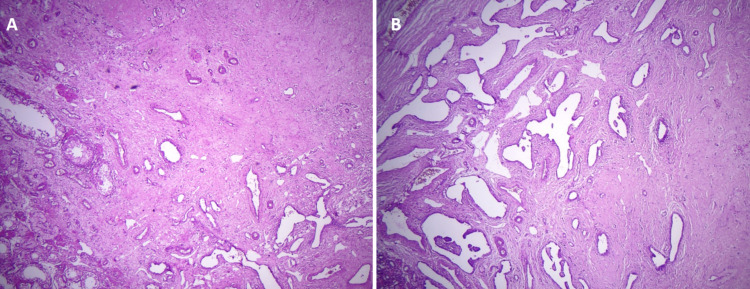
Microscopic examination of right testis shows two areas of scar formation, with one fibrotic area near the rete testis. There is no evidence of neoplastic cells, with only scar tissue remnant of a burned-out “Azzopardi” GCT identified: (A) ×10 and (B) ×20.

The patient’s CT scan of the abdomen and pelvis showed a large right retroperitoneal mass causing lateral displacement of the kidney (Figure 1). The initial radiographic characteristics of the tumor were highly suggestive of a primary retroperitoneal sarcoma versus a less likelihood of renal cell carcinoma (RCC). A scrotal ultrasound was obtained because of the patient’s complaint of right groin pain, which showed two small hypoechoic foci in the right testicle (Figure 2). The patient’s testicular tumor markers revealed an elevated β-human chorionic gonadotropin (β-hCG) and lactate dehydrogenase (LDH) but a normal α-fetoprotein (AFP). The patient underwent a right radical orchiectomy. The gross examination of the right testis showed two foci of scar tissue with no evidence of viable malignant tissue. On microscopic examination, the right testis showed fibrotic regions without any evidence of viable neoplastic cells, consistent with a burned-out (Azzopardi) testicular tumor (Figure 3).

**Figure 4:**
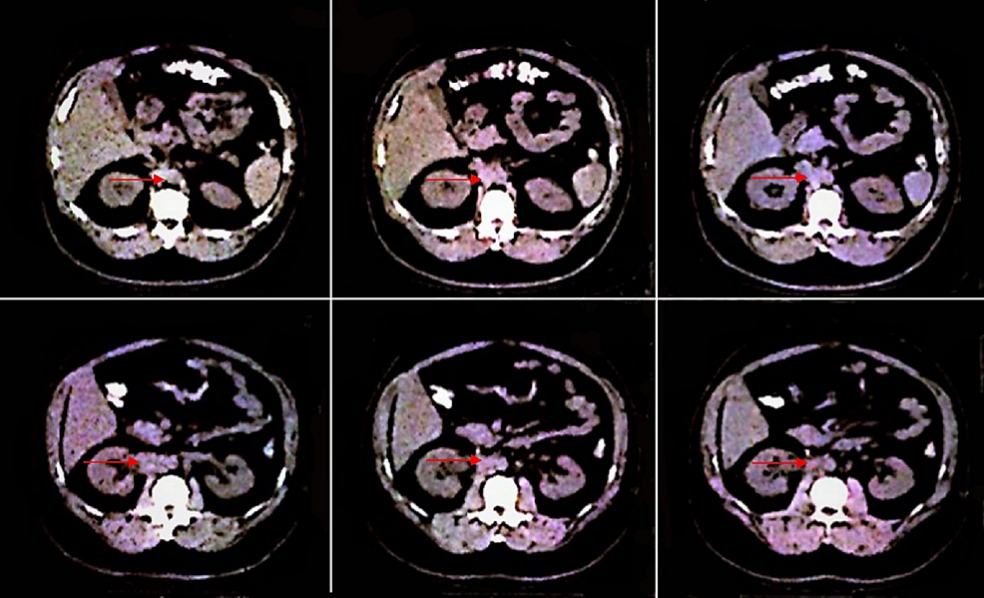
CT-scan of the abdomen and pelvis after four cycles of chemotherapy with BEP showed a significantly smaller yet persistently enhancing retroperitoneal mass. CT: computed tomography. BEP: bleomycin, etoposide, and cisplatin. Red arrows point to the remaining retroperitoneal mass post-chemotherapy.

**Figure 5:**
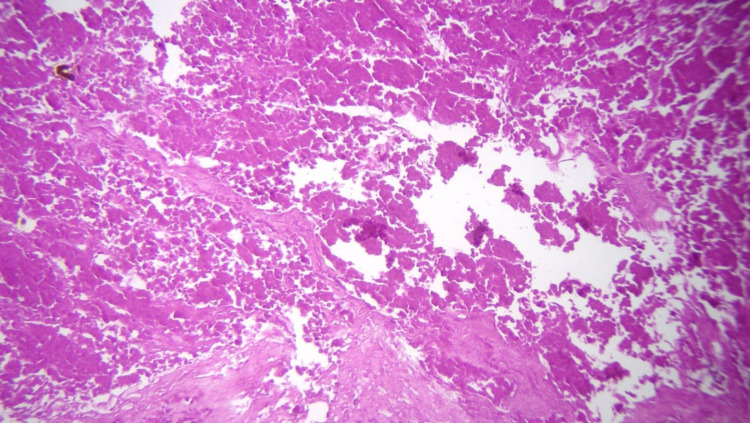
Microscopic examination of patient’s lymph node after chemotherapy shows extensive necrosis without viable neoplastic cells (×20).

Subsequently, the patient had a CT scan of the chest and the mediastinum, showing no metastatic disease. A biopsy of the retroperitoneal mass revealed testicular germ cell neoplasia. Post-orchiectomy tumor markers showed a mild decrease in β-hCG and LDH, although both remained significantly high compared to the normal range. The patient received four cycles of chemotherapy with bleomycin 30 mg, etoposide 200 mg, and cisplatin 50 mg (BEP) without any treatment-related complications. After completing the entire course of BEP, the patient’s testicular tumor markers, including β-hCG and LDH, were within normal limits. His abdomen and pelvis CT scans, however, showed a persistently enhancing retroperitoneal mass (Figure 4). The patient underwent RPLND with the removal of 12 para-aortic and paracaval lymph nodes. Microscopic examination showed a single lymph node with extensive necrosis, with other lymph nodes showing no abnormalities or evidence of malignancy (Figure 5). The patient’s abdomen and pelvis CT scans 12 months later showed no evidence of the retroperitoneal mass. The patient was followed-up every six months afterward for five years without any evidence of disease recurrence.

## Discussion

Burned-out testicular GCTs are extra-gonadal metastatic GCTs with no evidence of neoplastic cells in the testes [6]. The first case of a burned-out testicular tumor was reported by Prym et al. in 1927 of a deceased 51-year-old man with multi-organ metastatic disease with chorionic epithelial histology [7]. Only scar tissue was found in the right testicle, with no identifiable neoplastic cells. Prym attributed these findings to the spontaneous regression of the primary testicular tumor [7]. While the exact pathogenesis of burned-out GCTs remains elusive, two significant hypotheses have emerged as being responsible for the burnout phenomenon. The first theory suggests that the primary testicular GCT undergoes spontaneous regression after metastasizing, either because of an immune response or tumor ischemia [8-10]. The other theory proposes the de-novo formation of a primary GCT in extra-gonadal tissues [11,12]. In our patient, the first theory of spontaneous regression is the more likely scenario, as his right testis had two foci of scar formation on pathologic exam. These scars can only be explained by the spontaneous regression theory, with a possible immune response or ischemia causing spontaneous regression of the primary testicular GCT.

Burned-out testicular tumors are generally difficult to diagnose, as there is no evidence of primary testicular cancer. This often leads to the misdiagnosis of the metastatic lesions as the primary tumor [13]. This was also the case in our patient, whose initial abdomen and pelvis CT scans were highly suggestive of a primary retroperitoneal sarcoma. When burned-out GCT is suspected, high-resolution testicular ultrasound is the best diagnostic study, as it can detect small, highly echogenic foci, as well as microlithiasis or microcalcifications, all of which are sonographic hallmarks of burned-out tumors [13,14]. A positron emission tomography (PET) scan is often non-diagnostic, as it may show normal uptake in the testes because there are no viable neoplastic tissues. Therefore, ultrasonography is the gold-standard imaging modality for diagnosing burned-out testicular GCTs [13].

Differentiation between burned-out testicular tumors and primary extra-gonadal GCTs is crucial, as it can change the patient’s treatment plan and prognostic outcome. Primary testicular cancers are more sensitive to chemoradiation and typically have a significantly better outcome, with a more than 90% five-year survival rate [4,11]. Meanwhile, extra-gonadal NSGCTs are often more resistant to chemoradiation and have a significantly lower five-year survival rate of approximately 65% [4,12]. Therefore, the possibility of burned-out GCTs in men presenting with a retroperitoneal or mediastinal tumor should always be considered, despite the absence of testicular nodules on the physical exam.

## Conclusions

Burned-out testicular tumors are a rare clinical phenomenon and are often difficult to diagnose. Here, we report the rare case of a patient with a burned-out testicular tumor who presented with a large retroperitoneal mass. The initial radiographic findings of the mass were highly suggestive of primary retroperitoneal sarcoma versus a significantly lower likelihood of metastatic RCC. However, because of his initial complaint of right groin pain, we obtained an ultrasound of his testicles, which revealed two hypoechoic foci, which were found to be burned-out testicular tumors after right orchiectomy. The patient was then successfully treated with four cycles of BEP, remaining disease-free after five years of follow-up. This report highlights the importance of considering burned-out testicular tumors in patients presenting with a retroperitoneal mass, despite the absence of testicular mass. Given the potential for testicular tumors to undergo spontaneous regression after metastasis, it is prudent for clinicians to obtain a testicular ultrasound in young and middle-aged men presenting with a retroperitoneal mass.
